# Hematoma Shape, Hematoma Size, Glasgow Coma Scale Score and ICH Score: Which Predicts the 30-Day Mortality Better for Intracerebral Hematoma?

**DOI:** 10.1371/journal.pone.0102326

**Published:** 2014-07-16

**Authors:** Chih-Wei Wang, Yi-Jui Liu, Yi-Hsiung Lee, Dueng-Yuan Hueng, Hueng-Chuen Fan, Fu-Chi Yang, Chun-Jen Hsueh, Hung-Wen Kao, Chun-Jung Juan, Hsian-He Hsu

**Affiliations:** 1 Department of Radiology, Tri-Service General Hospital and National Defense Medical Center, Taipei, Taiwan, Republic of China; 2 Department of Automatic Control Engineering, Feng Chia University, Taichung, Taiwan, Republic of China; 3 Department of Neurological Surgery, Tri-Service General Hospital and National Defense Medical Center, Taipei, Taiwan, Republic of China; 4 Department of Pediatrics, Tri-Service General Hospital and National Defense Medical Center, Taipei, Taiwan, Republic of China; 5 Department of Neurology, Tri-Service General Hospital and National Defense Medical Center, Taipei, Taiwan, Republic of China; The George Washington University, United States of America

## Abstract

**Purpose:**

To investigate the performance of hematoma shape, hematoma size, Glasgow coma scale (GCS) score, and intracerebral hematoma (ICH) score in predicting the 30-day mortality for ICH patients. To examine the influence of the estimation error of hematoma size on the prediction of 30-day mortality.

**Materials and Methods:**

This retrospective study, approved by a local institutional review board with written informed consent waived, recruited 106 patients diagnosed as ICH by non-enhanced computed tomography study. The hemorrhagic shape, hematoma size measured by computer-assisted volumetric analysis (CAVA) and estimated by ABC/2 formula, ICH score and GCS score was examined. The predicting performance of 30-day mortality of the aforementioned variables was evaluated. Statistical analysis was performed using Kolmogorov-Smirnov tests, paired t test, nonparametric test, linear regression analysis, and binary logistic regression. The receiver operating characteristics curves were plotted and areas under curve (AUC) were calculated for 30-day mortality. A P value less than 0.05 was considered as statistically significant.

**Results:**

The overall 30-day mortality rate was 15.1% of ICH patients. The hematoma shape, hematoma size, ICH score, and GCS score all significantly predict the 30-day mortality for ICH patients, with an AUC of 0.692 (*P* = 0.0018), 0.715 (*P* = 0.0008) (by ABC/2) to 0.738 (*P* = 0.0002) (by CAVA), 0.877 (*P*<0.0001) (by ABC/2) to 0.882 (*P*<0.0001) (by CAVA), and 0.912 (*P*<0.0001), respectively.

**Conclusion:**

Our study shows that hematoma shape, hematoma size, ICH scores and GCS score all significantly predict the 30-day mortality in an increasing order of AUC. The effect of overestimation of hematoma size by ABC/2 formula in predicting the 30-day mortality could be remedied by using ICH score.

## Introduction

Hematoma shape has been recently reported to be related to hematoma size and hematoma growth of intracerebral hematomas (ICH). An irregular shape of hematoma has been linked to a larger hematoma size [1] and a higher risk of hematoma growth [2], which in turns is related to a poorer outcome [3]. Whether the hematoma shape predicts the 30-day mortality for ICH patients is of clinical interest and importance. It has not been reported in this regard, however.

Hematoma size is an important prognostic factor predicting the mortality of ICH patients [4]–[5]. The hematoma size could be either estimated simply by the ABC/2 formula or measured by computed assisted volumetric analysis (CAVA). Although the ABC/2 formula has been used to estimate the hematoma size for predicting clinical outcomes, it tends to overestimate the hematoma size as compared to the CAVA by 2% to 32% [6]–[7]. It remains unanswered whether the estimation error of hematoma size leads to imprecision in predicting the 30-day mortality of ICH patients.

By weighting Glasgow coma scale (GCS) score, age, hematoma size, origin of ICH, and ventricular involvement, the ICH score has been raised as a simple method in predicting 30-day mortality [8]. However, the performance of the ICH score in predicting the 30-day mortality varies in different studies, with the areas under the curve (AUC) in the receiver operating characteristics (ROC) curves ranging from 0.72 to 0.92 [8]–[11]. Whether the ICH score predicts the 30-day mortality better than the hematoma shape, hematoma size, and GCS score has not been assessed in a single study yet.

In this study, we aimed to investigate the performance of hematoma shape, hematoma size, GCS score, and ICH score in predicting the 30-day mortality for ICH patients. In the meanwhile, we also examined the influence of the estimation error of hematoma size on the prediction of 30-day mortality.

## Materials and Methods

This retrospective study was approved by the institutional review board of Tri-Service General Hospital (1-103-05-014). The institutional review board waived the need of written informed consent from the patients in this study. All data underlying the findings described in this manuscript are in Table S1.

### Subjects

Brain computed tomography (CT) images of 137 patients, who were admitted to our hospital under the diagnosis of acute intracerebral hemorrhage during a period of 10 months, were initially reviewed. All patients received non-enhanced CT with an interval between the time of CT study and the onset of symptoms within 24 hours. Twenty-three patients were excluded based on the following exclusion criteria including concurrent subarachnoid hemorrhage (N = 4), subdural hematoma (N = 1), brain tumor (N = 2), acute ischemic infarction with hemorrhagic transformation (N = 3), old ischemic infarction (N = 5), old hemorrhage (N = 2), severe motion artifact (N = 2), beam hardening artifact (N = 1), and recent intracranial operations (N = 3). Eight patients who had a ventricular shunting catheter seen on CT images were also excluded. Finally, 106 patients (62.8±15.3 years, M: F = 1.9: 1) with intracerebral hematomas were recruited in this study. The clinical data (gender, age, GCS score, blood pressure, pulse rate, respiratory rate, and body temperature), clinical history (hypertension, diabetes mellitus, ischemic heart disease, and congestive heart failure), and laboratory data (blood sugar, white blood cell count, hemoglobin, platelet count, and international normalized ratio) obtained on admission were recorded. To assess the 30-day mortality, the survival statuses of patients were confirmed based on the medical records not only on admission but also in outpatient follow up.

### Imaging data acquisition

Non-enhanced CT images were acquired using single or multi-row spiral CT scanners (Somatom Plus 4, Simens, Erlangen, Germany; PQ6000, Picker International Inc., Highland Heights, Ohio, U.S.A.; Brilliance, Philips Medical System, Cleveland, Ohio, U.S.A), which were compatible with the regulation of digital imaging and communications in medicine (DICOM). Imaging parameters included 140 kV, 170 mA, slice thickness of 5 mm, slice gap of 0 mm, field of view of 210 mm, matrix size of 512×512 and gantry angulation being parallel to the supraorbitomeatal line.

### Qualitative analysis of hematoma shape

**Figure 1:**
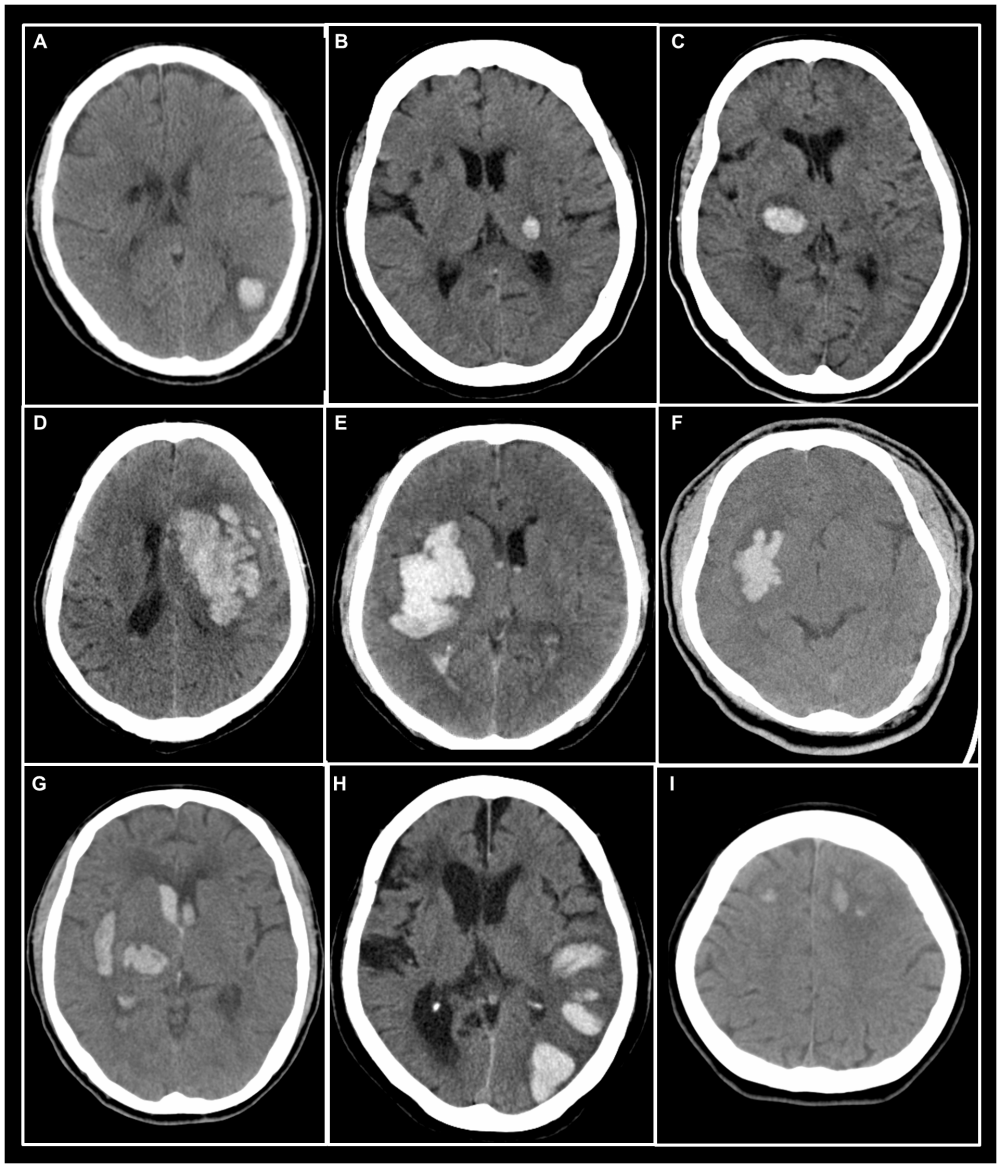
Axial brain CT images demonstrating regular hematoma shapes (A–C) and irregular hematoma shapes (D–I). Regular hematoma shape implies roundish (A) or ovoid (B & C) shape with smooth margin, while irregular hematoma shape refers to pleomorphic contour (D–F), separated adjacent hematomas (G & H) and multicentric hematomas (I).

The hematoma shape was independently evaluated by two neuroradiologists (observer 1, C.W.W.; observer 2, C.J.J.) using a binary scoring system, which was first introduced here in this study. A score of 0 represented a regular hematoma shape, including a roundish or ellipsoid hematoma with smooth margin (Fig. 1A–C). A score of 1 represented an irregular hematoma shape, including a pleomorphic contour of hematoma (Fig. 1D–F), several adjacent but separated hematomas (Fig. 1G & H) and multicentric hematomas (Fig. 1I).

### Data analysis and volumetric analysis of ICH

The DICOM data were firstly stored in the picture archiving and communication system (PACS) immediately after acquisition. Maximal diameters of the hemorrhage in three orthogonal directions were measured on a PACS viewer (EBM Technologies Inc., Taipei, Taiwan) by a 10-year experienced neuroradiologist (C.J.J.) for calculating the hemorrhagic size using the ABC/2 method as described by Kothari et al. [6].CT image data were also digitally transferred to a personal computer for hemorrhagic volumetric analysis processed with the software programs developed (by Y.J.L.) using Matlab (MathWorks, Natick, MA, U.S.A.). All image processing, region-of-interest drawing, and data analyses were performed by a single author (C.J.J.). The data processing included histogram analysis of a large region of interest (ROI), threshold setting for the hemorrhage, manually polygonal ROI drawing, voxel counting and size calculation slice-by-slice, and finally summing hemorrhagic size of each slice. Thresholds with a lower level of 40∼45 HU and an upper level of 100∼150 HU were adjusted as a mask for the hemorrhage as described by Strik et al. [12]. Small polygonal ROIs encompassing the hyperdense hemorrhage were drawn to avoid the contamination of noises from the adjacent gray matter. The hematoma size was calculated by the equation:
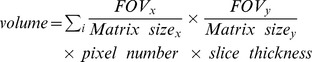



### ICH Score and 30-day mortality

The ICH score was calculated based on the clinical information (age and GCS score) and the CT features (hematoma size, infratentorial origin of hemorrhage, and intraventricular hemorrhage) as developed by Hemphill et al [8]. The 30-day mortalities were calculated with respect to the hematoma shape, hematoma size, ICH Score, and other parameters used in calculating ICH Score.

### Statistical analysis

Statistical analyses were performed using SPSS Version 16.0 software (SPSS Inc, Chicago, III) and MedCalc Version 13.0 (MedCalc Software Inc, Ostend Belgium). The interobserver reliability was examined by linearly weighted kappa statistics. The normality of parameters was examined by using Kolmogorov-Smirnov tests. Cross-tabulations were analyzed using Fisher exact test. Paired student t test was used for 2-group comparisons regarding parameters with normal distribution, while nonparametric tests (Wilcoxon signed rank tests and McNemar tests) were used for 2-group comparisons regarding parameters without normal distribution. Linear regression analysis was used to assess the relationship between the hematoma size estimated by ABC/2 formula and the hematoma size measured by CAVA. Binary logistic regression was applied to examine the relationships between the independent variables (shape, size, GCS score, age, presence of IVH, presence of infratentorial location) and dependent variable (30-day mortality). The nonparametric receiver operating characteristics (ROC) curves were plotted and areas under curve (AUC) were calculated for 30-day mortality. A P value less than 0.05 was considered as statistically significant.

## Results

Of 106 patients, 77 (73%) had a history of hypertension, 21 (20%) had type II diabetes mellitus, four (3.8%) had impaired coagulation function with international normalized ratio (INR) ≧ 1.5, and 47 (44.3%) had simultaneous IVH. The hematomas were located supratentorially in 93 (87.7%) patients, located infratentorially in 13 (12.2%) patients. The overall 30-day mortality was 15.1% (16 of 103). The demographic and clinical characteristics of our study were summarized in Table 1. Factors that were significantly associated with outcomes in 30 days included platelet count (*P* = 0.003), hematoma shape (*P* = 0.003), hematoma size (*P* = 0.009), presence of IVH (*P*<0.032), GCS score (*P*<0.001), and ICH score (*P*<0.001).

**Table 1:** Demographic and clinical characteristics of ICH patients.

	Variable	Death* (n = 16)	Survival* (n = 90)	Total (n = 106)	*P* value	Missing data
	Males	11 (68.8%)	58 (64.4%)	69 (65.1%)	0.74	0
	Age (years)	62.1±16.1	63.0±15.2	62.8±15.3	0.83	0
	Age ≧ 80 years	3 (18.8%)	16 (17.8%)	19 (17.9%)	0.92	0
**Vital signs**	Systolic BP (mmHg)	185.3±2.3	171.6±34.3	173.7±35.7	0.23	0
	Diastolic BP (mmHg)	96.6±14.3	91.3±18.7	9.1±18.1	0.21	0
	Pulse pressure (mmHg)	88.7±30.7	80.3±25.3	81.6±26.2	0.31	0
	Pulse rate (per minute)	91.8±14.8	89.7±12.3	90.0±12.7	0.60	0
	Respiratory rate (per minute)	20.1±1.7	19.7±1.9	19.7±1.8	0.44	0
	Body temperature (°C)	36.7±0.3	36.8±0.4	36.8±0.4	0.12	0
**Laboratory data**	Glucose (mg/dL)	185.6±89.5	145.4±51.3	149.5±57	0.22	18 (17.0%)
	WBC count (×10^3^/µL)	11.9±5.3	10.5±3.9	10.7±4.1	0.34	3(2.8%)
	Hemoglobin (mg/dL)	12.2±2.8	13.3±2.4	13.1±2.5	0.16	1 (0.9%)
	Platelet count (×10^5^/µL)	1.76±0.83	2.53±0.99	2.43±1.0	0.003	1 (0.9%)
	INR	1.4±0.5	1.2±1.0	1.2±1.0	0.43	19 (18.0%)
**CT findings**	Irregular shape	12 (75%)	33 (36.7%)	45 (42.5%)	0.004	0
	Hematoma size (mL) (CAVA)	62.7±57.2	21.6±26.2	27.8±35.6	< 0.001	0
	Hematoma size (mL) (ABC/2)	90.3±20.1	29.5±4.0	38.7±51.0	0.009	0
	Hematoma size >30 mL (CAVA)	11 (68.8%)	19 (21.1%)	30 (28.3%)	0.001	0
	Hematoma size >30 mL (ABC/2)	11 (68.8%)	24 (26.7%)	35 (33.0)	0.001	0
	Intraventricular hemorrhage	11 (68.8%)	36 (40%)	47 (44.3%)	0.032	0
	Infratentorial origin	3 (18.8%)	10 (11%)	13 (12.3%)	0.48	0
	GCS score	4.4±2.1	10.8±3.9	9.8±4.3	<0.001	0
	ICH score	3.6±1.0	1.5±1.3	1.8±1.5	<0.001	0

Note: Data are presented as mean ± standard deviation or numbers (%); BP: blood pressure; GCS: Glasgow coma scale; ICH: intracerebral hematoma; INR: international normalized ratio;

*Patients are classified into death and survival groups based on the 30-day mortality.

### Location of hematomas

The 30-day mortality was 23.1% (3 of 13) for infratentorial hematomas and was 14.0% (13 of 93) for supratentorial hematomas. The difference between the aforementioned locations, however, was not statistically significant (*P* = 0.391). Patients with pontine hematomas had a 30-day mortality as high as 33.3% (2 of 6), while patients with cerebellar hematomas had a 30-day mortality of 14.3% (1 of 7). Again, the difference was not statistically significant (*P* = 0.279).

### Shape of hematomas: regular hematomas versus irregular hematomas

Hematomas were judged as regular in 62 (58.5%) patients and irregular in 44 (41.5%) patients by observer 1, while regular in 61 (57.5%) patients and irregular in 45 (42.5%) patients by observer 2. Ten patients whose hematomas were judged as regular by observer 1 were considered as irregular by observer 2. On the other hand, 8 patients whose hematomas were judged as irregular by observer 1 were considered as regular by observer 2. Linear weighted Kappa statistics showed a weighted kappa value of 0.632 with a confidence level from 0.483 to 0.782, suggestive of substantial agreement.

The differences between regular hematomas and irregular hematomas were listed in Table 2. The irregular group did not differ from regular group in gender (*P* = 1) and age (*P* = 0.92). Regarding the vital signs, the irregular group had a significantly higher diastolic pressure (*P* = 0.021) and higher pulse rate (*P* = 0.022) than the regular group. The laboratory data showed that the irregular group had a significantly higher WBC count (*P* = 0.003) than the regular group. With respect to the consciousness, the irregular group had a significantly lower GCS score than regular group (*P*<0.001). Taking the CT imaging features into consideration, the irregular group had a significantly larger hematoma size (*P*<0.001), significantly higher percentage of hematomas larger than 30 mL (*P*<0.001), and significantly higher ratio with intraventricular hemorrhage (*P*<0.001). The ICH score was significantly higher on irregular group than the regular group (*P*<0.001). The 30-day mortality was also significantly higher on irregular group than the regular group (*P*<0.006).

**Table 2:** Comparisons between regular and irregular intracerebral hematomas.

	Hematoma shape	Regular (n = 61)	Irregular (n = 45)	*P* value
	Males	21 (34.4%)	16 (35.6%)	1
	Age (years)	62.7±15.0	63.0±15.9	0.92
	Age ≧ 80 years (%)	10 (16%)	9 (20%)	0.798
**Vital signs**	Systolic blood pressure (mmHg)	168.4±31.0	180.9±40.5	0.088
	Diastolic blood pressure (mmHg)	88.5±15.6	97.0±20.3	0.021
	Pulse pressure (mmHg)	79.9±22.8	83.9±30.4	0.46
	Pulse rate (beat per minute)	87.4±9.7	93.5±15.2	0.022
	Respiratory rate (per minute)	19.7±1.3	19.8±2.4	0.81
	Body temperature (°C)	36.8±0.4	36.8±0.2	0.70
**Laboratory data**	Blood sugar (mg/dL)	143.3±51.4	158.1±63.5	0.25
	WBC count (×10^3^/µL)	9.6±3.7	12.2±4.3	0.003
	Hemoglobin (mg/dL)	13.4±2.2	12.8±2.7	0.23
	Platelet count (×10^5^/µL)	2.46±0.94	2.37±1.09	0.67
	INR	1.26±1.27	1.17±1.27	0.66
**CT findings**	Hematoma size* (mL)	9.7±10.5	52.3±42.5	<0.001
	Hematoma size*>30 mL	3 (4.9%)	27 (60%)	<0.001
	Intraventricular hemorrhage	26	69	<0.001
	Infratentorial origin	9 (14.8%)	4 (8.9%)	0.551
	GCS score	11.7±3.7	7.2±3.7	<0.001
	ICH score	1.1±1.2	2.8±1.2	<0.001
	30-day mortality rate (%)	4 (6.6%)	12 (26.7%)	<0.006

Note: Data are presented as mean ± standard deviation or numbers (%); INR: international normalized ratio; GCS: Glasgow coma scale; ICH: intracerebral hematoma;

*Hematoma size is measured by computer-assisted volumetric analysis.

### Size of hematoma: ABC/2 formula versus CAVA

**Figure 2:**
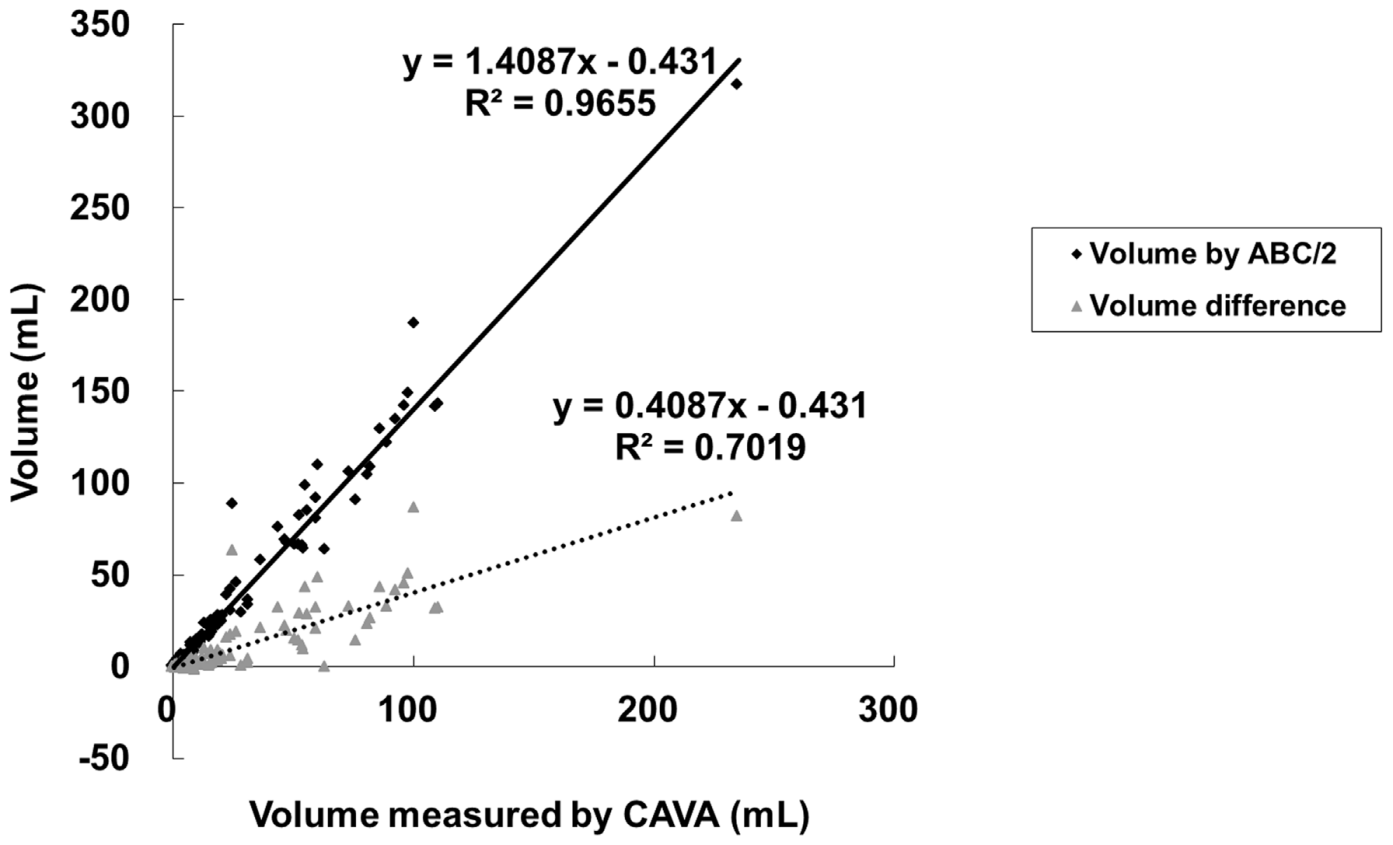
Scatter plots of size estimated by ABC/2 formula and size difference versus size measured by CAVA of intracerebral hematomas.

The overall hematoma size estimated by the ABC/2 formula (38.7±51.0 ml) was significantly higher than that measured by the CAVA (27.8±35.6 ml) (P<0.001). Linear regression analysis showed that the hematoma size (y) estimated by ABC/2 formula was positively correlated to the hematoma size (x) measured by CAVA characterized by the equation, y = 1.409x–0.431, with a R^2^ of 0.965 significantly (*P*<0.001). The size difference (y) between two methods was also positively correlated to the hematoma size (x) measured by CAVA characterized by the equation, y = 0.409x–0.434, with a R^2^ of 0.702 significantly (*P*<0.001) (Fig. 2).

### GCS score

The overall GCS score was 9.8±4.3. Nineteen patients (17.9%) had a GCS score of 3 or 4, 45 patients (42.4%) had a GCS score ranging 5 to 12, while 42 patients (39.6%) had a GCS score ranging from 13 to 15. Patients with a GCS score of 3 to 4 had a significantly higher 30-day mortality (68.4%) than those with a GCS score of 5 to 12 (6.7%) and a GCS score of 13 to 15 (0%).

### ICH Score

**Figure 3:**
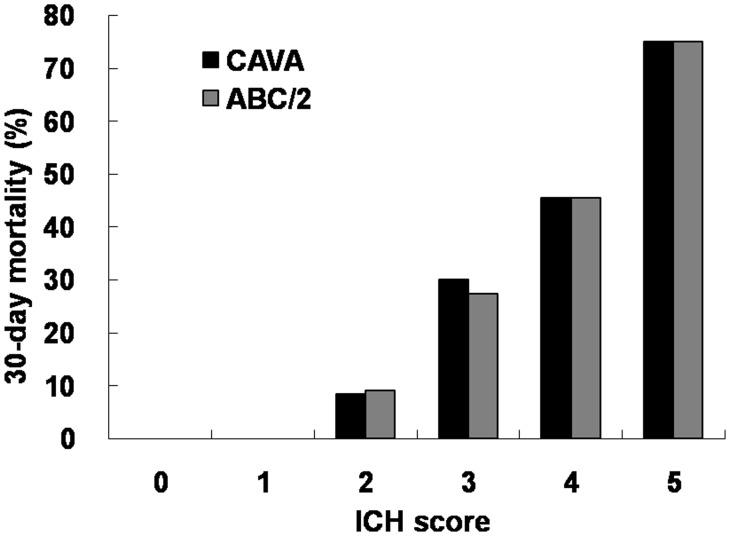
Bar chart of 30-day mortality versus ICH Score. There is no apparent difference between the ABC/2 and CAVA methods regarding the 30-day mortality no matter in trend or in each ICH score.

The relationship of 30-day mortality versus ICH score was shown on Fig. 3. There was a positive association between the 30-day mortality and ICH score, i.e., with a higher ICH score, the 30-day mortality was also higher, no matter the hematoma size was estimated by ABC/2 formula or measured by CAVA. In each ICH score, the 30-day mortality was of no significant difference between the ABC/2 group and CAVA group.

### Receiver operating characteristic (ROC) curves

**Figure 4:**
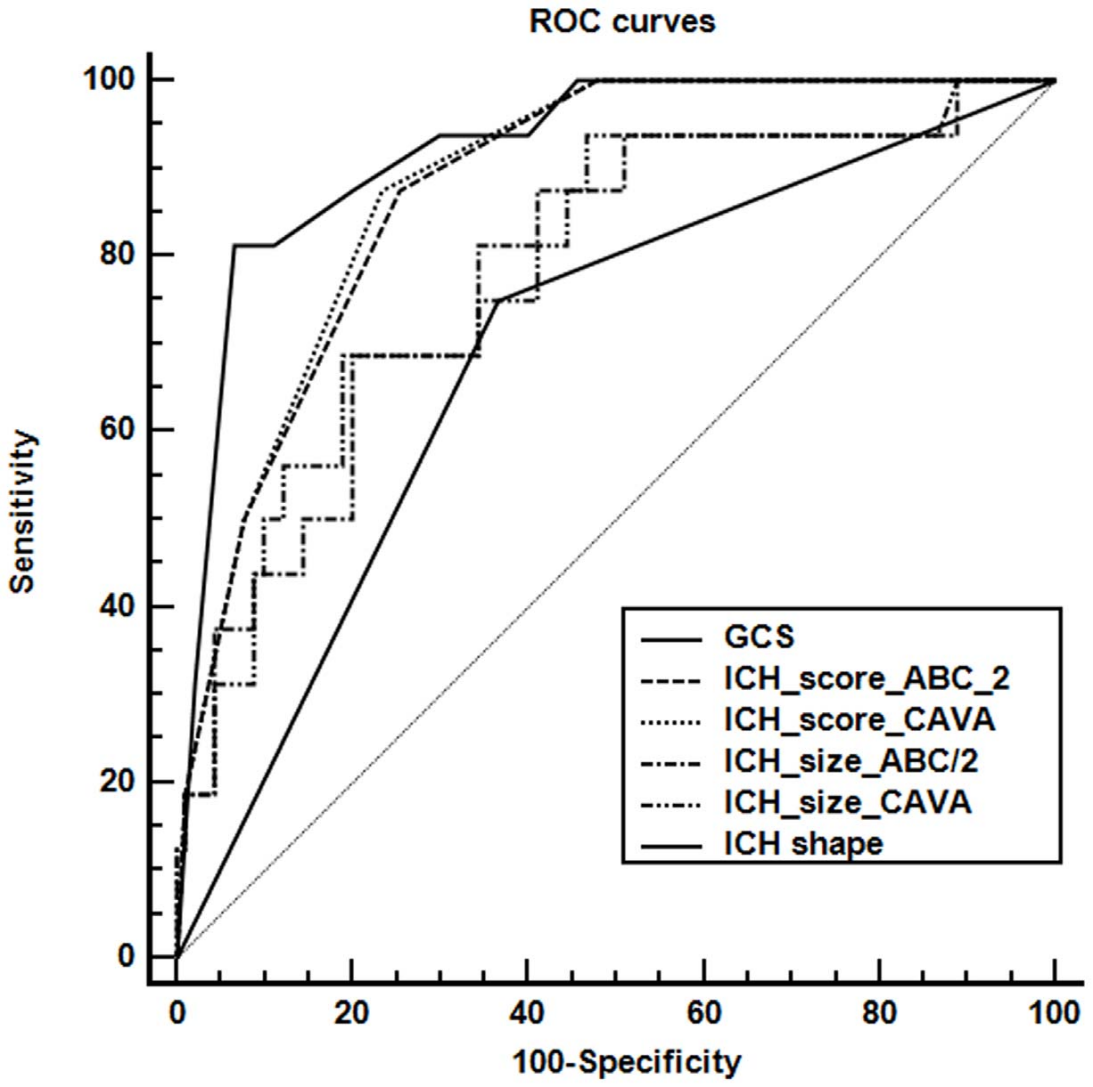
Comparison of ROC curves in predicting the 30-day mortality by GCS, ICH scores, ICH size, and ICH shape.

Comparison of ROC curves in predicting the 30-day mortality by GCS, ICH scores, ICH size, and ICH shape was graphically demonstrated (Fig. 4). The predicting performances of the variables were summarized in Table 3. The variables of hematoma shape, hematoma size, ICH score, and GCS score all significantly predicted the 30-day mortality with the AUC of 0.692 (*P* = 0.0018), 0.715∼0.786 (*P*≤0.0008), 0.877∼0.882 (*P*<0.0001), and 0.912∼0.922 (*P*<0.0001), respectively. Pairwise comparisons of ROC curves, summarized in Table 4, showed significant differences between AUCs predicted by hematoma size measured by CAVA and hematoma size estimated by ABC/2 formula (*P* = 0.022), predicted by GCS score and hematoma size (*P* = 0.0145∼0.0048), predicted by ICH score and hematoma size (*P* = 0.0336∼0.0076), predicted by GCS score and hematoma shape (*P* = 0.0009), and predicted by ICH score and hematoma shape (*P* = 0.0012∼0.0076). The difference between AUCs predicted by ICH score in which the hematoma size was estimated by ABC/2 formula and AUCs predicted by ICH score in which the hematoma size was measured by CAVA was not statistically significant (*P* = 0.11). Likewise, the difference between AUCs predicted by ICH score and predicted by GCS score was also not statistically significant (*P* = 0.14∼0.24).

**Table 3:** Predictive performance of the variables.

	Criterion	AUC	Sn (%)	Sp (%)	LR+	LR-	Youden index	*P value*
GCS score*	>1	0.912	81.25	92.22	10.5	0.2	0.7347	<0.0001
ICH score (CAVA)	>2	0.882	87.5	76.67	3.75	0.2	0.6417	<0.0001
ICH score (ABC/2)	>2	0.877	87.5	74.44	3.42	0.2	0.6194	<0.0001
ICH size (CAVA)*	>0	0.738	68.75	78.89	3.26	0.4	0.4764	0.0002
Platelet (/µL)	≤167000	0.722	53.33	89.77	5.21	0.5	0.4311	0.0023
ICH size (ABC/2)*	>0	0.715	68.75	74.16	2.66	0.4	0.4291	0.0008
ICH shape	>0	0.692	75	63.33	2.05	0.39	0.3833	0.0018
IVH*	>0	0.644	68.75	60	1.72	0.5	0.2875	0.0275
WBC (/µL)	>11900	0.568	53.33	69.32	1.74	0.67	0.2265	0.444
Infratentorial origin*	>0	0.538	18.75	88.89	1.69	0.9	0.07639	0.4717

AUC: Area under the receiver operating characteristics curve; Sn: Sensitivity; Sp: Specificity; LR+: positive likelihood ratio; LR-: negative likelihood ratio; GCS: Glasgow coma scale; ICH: intracerebral hematoma; CAVA: computer-assisted volumetric analysis; IVH: intraventricular hemorrhage;

*Indicates a parameter used in the ICH score [8].

**Table 4:** Pairwise comparisons of ROC curves.

Pairwise comparison	Difference between AUCs	Standard error	95% CI	Z statistic	*P*-value
ICH size (ABC/2) versus ICH shape	0.0193	0.0598	−0.0979 to 0.136	0.323	0.7467
ICH size (CAVA) versus ICH shape	0.0474	0.0597	−0.0696 to 0.164	0.794	0.4272
GCS score versus ICH score (CAVA)	0.0277	0.0238	−0.0189 to 0.0744	1.166	0.2438
GCS score versus ICH score (ABC/2)	0.0362	0.025	−0.0128 to 0.0851	1.447	0.1478
ICH score (ABC/2) versus ICH score (CAVA)	0.00843	0.00528	−0.00192 to 0.0188	1.596	0.1104
ICH score (ABC/2) versus ICH size (CAVA)	0.133	0.0628	0.0103 to 0.257	2.124	0.0336
ICH score (CAVA) versus ICH size (CAVA)	0.142	0.0626	0.0192 to 0.265	2.266	0.0234
ICH size (CAVA) versus ICH size (ABC/2)	0.0281	0.0123	0.0040 to 0.052	2.289	0.0221
GCS score versus ICH size (CAVA)	0.17	0.0694	0.0336 to 0.306	2.444	0.0145
ICH score (ABC/2) versus ICH size (ABC/2)	0.162	0.0632	0.0377 to 0.285	2.557	0.0106
ICH score (CAVA) versus ICH size (ABC/2)	0.17	0.0637	0.0452 to 0.295	2.669	0.0076
GCS score versus ICH size (ABC/2)	0.198	0.0702	0.0602 to 0.335	2.818	0.0048
ICH score (ABC/2) versus ICH shape	0.181	0.0556	0.0718 to 0.290	3.251	0.0012
GCS score versus ICH shape	0.217	0.0651	0.0894 to 0.345	3.333	0.0009
ICH score (CAVA) versus ICH shape	0.189	0.0561	0.0792 to 0.299	3.372	0.0007

ROC: receiver operating characteristics; AUC: Area under the ROC curve; CI: confidence interval; ICH: intracerebral hematoma; CAVA: computer-assisted volumetric analysis; GCS: Glasgow coma scale.

## Discussion

ICH shape is getting increasing attention recently. Compared to the regular hematoma, the irregular hematoma has been related to a higher risk of hematoma growth [2], which in turns serves a predictor of poor outcome [3] and a determinant of mortality [13]. Nevertheless, whether the hematoma shape independently predicts the 30-mortality has not been verified yet. Our results show that the irregular hematoma has significantly larger hematoma size (52.3 mL versus 9.7 mL) (*P*<0.001) and higher ratio of hematoma size which is more than 30 mL (60% versus 4.9%) (*P*<0.001). In addition to hematoma size, the irregular hematoma is also associated with significantly higher ratio of IVH (69% versus 26%) (*P*<0.001), lower GCS score (7.2 versus 11.7) (*P*<0.001), and higher ICH score (2.8 versus 1.1) (*P*<0.001). The irregular hematoma has significantly higher 30-day mortality rate (26.7% versus 6.6%) than the regular hematoma (*P*<0.006). ROC analysis discloses that the hematoma shape significantly predicts the 30-day mortality with an AUC of 0.692 (*P* = 0.0018). These associations between hematoma shape and the hematoma size, IVH, GCS score, and ICH score may explain why the hematoma shape serves as an independent predictor of the 30-day mortality.

Although the hematoma size has been proposed as an independent predictor of mortality and used in ICH score for ICH in some researches [4], [8], [14]–[15], it is not considered as an independent predictor for risk stratification and is discarded from ICH score calculation by other studies due to the concern of potential imprecision of hematoma size estimated by the ABC/2 formula [11], [16]–[17]. The ABC/2 formula has been found to overestimate the hematoma size in some studies [7], [18] but to underestimate the hematoma size in other studies [19]. Our study showed that the ABC/2 formula significantly overestimates the hematoma size up to 39.2%, which is higher than those reported by Huttner et al. (8.5%) and by Wang et al (28.7%) [18]. Our results further disclose that the hematoma size overestimated by the ABC/2 formula is positively and linearly related to the hematoma size measured by CAVA significantly (Fig 1). Using 30 mL as a criterion, the hematoma size estimated by the ABC/2 formula less accurately predicts the 30-day mortality (AUC  = 0.715) than the hematoma size measured by the CAVA (AUC  = 0.738) significantly (*P* = 0.02).

In predicting the 30-day mortality, the mean AUC of ICH scores in our study (0.882 using CAVA; 0.877 using ABC/2) are higher than Pengs' study (0.72) [9], Steins' study (0.736) [10], and Chuangs' study (0.74) [11], consistent with Clarkes' study (0.88) [20], and lower than Hemphrills' study (0.92%) [8]. Our study further reveals that the ICH score predicts the 30-day mortality superior to the hematoma shape (AUC  = 0.692) and the hematoma size (AUC  = 0.715 by ABC/2 and  = 0.738 by CAVA) with significant differences of AUC (*P* = 0.034 to 0.008). The AUC of ICH score with the hematoma size estimated by ABC/2 is not different to the AUC of ICH score with the hematoma size measured by CAVA.

Our study has three limitations. First, we did not evaluate the functional outcome or quality of life in this study. Nevertheless, 30-day mortality remains an important outcome in ICH patients and easy to be examined by neuroradiologists. Second, we didn't take surgical intervention into account in this study. According to a random trial performed by Auer et al, the mortality rate of patients who receive endoscopic aspiration of lobar intracerebral hemorrhage was significantly lower than those received medical treatment only [21]. Due to limited number of random trial, it is hard to get conclusion whether surgical intervention benefit ICH patients than medical treatment or not. Nevertheless, this important issue is beyond the scope of this study, in which only the initial conditions are taken into account. Third, our study recruited patients only from a single medical center in one country. The overall 30-day mortality rate (15.1%) of ICH in our study is consistent with those of other oriental population (15%∼20%) [8], [22] but is lower than in those of western countries (31.9%∼45%) [4], [6], [8], [14], [20], [22]–[24]. The difference of 30-day mortality rate between our study and the aforementioned western studies might come from racial difference, age difference, socioeconomic difference, et al. To eliminate the potential bias, a multi-national and multi-center study is suggested.

## Conclusions

Our study shows that hematoma shape, hematoma size, ICH scores and GCS score all significantly predict the 30-day mortality in an increasing order of AUC. Although the ABC/2 formula significantly overestimates the hemorrhagic size than the CAVA method, the effect of overestimation of hematoma size by ABC/2 formula in predicting the 30-day mortality could be remedied by using ICH score and GCS score.

## Supporting Information

Table S1 **Original data and ICH score of patients with intracerebral hematomas.**
(DOCX)
