# Supplementary material for: Hematoma Shape, Hematoma Size, Glasgow Coma Scale Score and ICH Score: Which Predicts the 30-Day Mortality Better for Intracerebral Hematoma?
Source: PLoS One. 2014 Jul 16;9(7):e102326. doi: 10.1371/journal.pone.0102326 (PMC4100880; doi:10.1371/journal.pone.0102326)
Supplement: Table S1 — Original data and ICH score of patients with intracerebral hematomas. (DOCX) [file pone.0102326.s001.docx]

Table S1. Original data and ICH score of patients with intracerebral hematomas

| no | sex | age | GCS | Volume (CAVA) | Volume (ABC/2) | Shape rater1 | Shape rater2 | expired | ICH score | | | | | | | |
| --- | --- | --- | --- | --- | --- | --- | --- | --- | --- | --- | --- | --- | --- | --- | --- | --- |
|  |  |  |  |  |  |  |  |  | age | location | Volume (CAVA) | Volume (ABC/2) | IVH | GCS | ICH score (CAVA) | ICH score (ABC/2) |
| 1 | F | 43 | 4 | 2.6 | 2.7 | 0 | 0 | 1 | 0 | 1 | 0 | 0 | 0 | 2 | 3 | 3 |
| 2 | F | 85 | 4 | 13.7 | 23.7 | 1 | 1 | 1 | 1 | 1 | 0 | 0 | 1 | 2 | 5 | 5 |
| 3 | M | 68 | 4 | 37.2 | 58.3 | 1 | 1 | 1 | 0 | 1 | 1 | 1 | 1 | 2 | 5 | 5 |
| 4 | M | 67 | 3 | 11.8 | 12.9 | 0 | 0 | 1 | 0 | 0 | 0 | 0 | 1 | 2 | 3 | 3 |
| 5 | F | 42 | 6 | 12.5 | 16.7 | 0 | 0 | 1 | 0 | 0 | 0 | 0 | 1 | 1 | 2 | 2 |
| 6 | M | 60 | 4 | 63.8 | 64.0 | 0 | 0 | 1 | 0 | 0 | 1 | 1 | 0 | 2 | 3 | 3 |
| 7 | F | 76 | 4 | 18.2 | 24.1 | 1 | 1 | 1 | 0 | 0 | 0 | 0 | 1 | 2 | 3 | 3 |
| 8 | M | 57 | 11 | 44.2 | 76.4 | 1 | 1 | 1 | 0 | 0 | 1 | 1 | 0 | 1 | 2 | 2 |
| 9 | F | 47 | 4 | 54.8 | 64.4 | 0 | 0 | 1 | 0 | 0 | 1 | 1 | 1 | 2 | 4 | 4 |
| 10 | M | 51 | 3 | 60.0 | 92.1 | 1 | 1 | 1 | 0 | 0 | 1 | 1 | 1 | 2 | 4 | 4 |
| 11 | M | 50 | 4 | 61.0 | 109.9 | 1 | 1 | 1 | 0 | 0 | 1 | 1 | 0 | 2 | 3 | 3 |
| 12 | M | 89 | 3 | 86.6 | 129.8 | 1 | 1 | 1 | 1 | 0 | 1 | 1 | 1 | 2 | 5 | 5 |
| 13 | F | 87 | 7 | 89.6 | 122.3 | 1 | 1 | 1 | 1 | 0 | 1 | 1 | 0 | 1 | 3 | 3 |
| 14 | M | 42 | 3 | 100.9 | 187.5 | 1 | 1 | 1 | 0 | 0 | 1 | 1 | 1 | 2 | 4 | 4 |
| 15 | M | 59 | 4 | 111.0 | 143.2 | 1 | 1 | 1 | 0 | 0 | 1 | 1 | 1 | 2 | 4 | 4 |
| 16 | M | 70 | 3 | 235.1 | 317.3 | 1 | 1 | 1 | 0 | 0 | 1 | 1 | 1 | 2 | 4 | 4 |
| 17 | M | 54 | 15 | 1.5 | 1.5 | 0 | 0 | 0 | 0 | 1 | 0 | 0 | 0 | 0 | 1 | 1 |
| 18 | F | 59 | 4 | 1.6 | 2.6 | 0 | 0 | 0 | 0 | 1 | 0 | 0 | 0 | 2 | 3 | 3 |
| 19 | F | 50 | 15 | 6.7 | 6.8 | 0 | 0 | 0 | 0 | 1 | 0 | 0 | 0 | 0 | 1 | 1 |
| 20 | M | 54 | 12 | 8.1 | 8.1 | 0 | 0 | 0 | 0 | 1 | 0 | 0 | 0 | 0 | 1 | 1 |
| 21 | F | 45 | 3 | 8.2 | 11.6 | 0 | 0 | 0 | 0 | 1 | 0 | 0 | 1 | 2 | 4 | 4 |
| 22 | M | 67 | 5 | 8.7 | 9.4 | 0 | 0 | 0 | 0 | 1 | 0 | 0 | 1 | 1 | 3 | 3 |
| 23 | M | 67 | 7 | 9.8 | 8.4 | 0 | 0 | 0 | 0 | 1 | 0 | 0 | 1 | 1 | 3 | 3 |
| 24 | F | 89 | 8 | 15.5 | 22.4 | 0 | 0 | 0 | 1 | 1 | 0 | 0 | 1 | 1 | 4 | 4 |
| 25 | F | 87 | 4 | 7.9 | 13.5 | 1 | 1 | 0 | 1 | 1 | 0 | 0 | 1 | 2 | 5 | 5 |
| 26 | M | 51 | 3 | 12.8 | 17.5 | 1 | 1 | 0 | 0 | 1 | 0 | 0 | 1 | 2 | 4 | 4 |
| 27 | F | 78 | 13 | 0.3 | 0.3 | 0 | 0 | 0 | 0 | 0 | 0 | 0 | 0 | 0 | 0 | 0 |
| 28 | M | 52 | 15 | 0.4 | 0.5 | 0 | 0 | 0 | 0 | 0 | 0 | 0 | 0 | 0 | 0 | 0 |
| 29 | M | 84 | 11 | 0.5 | 0.5 | 0 | 0 | 0 | 1 | 0 | 0 | 0 | 0 | 1 | 2 | 2 |
| 30 | F | 66 | 15 | 0.7 | 0.6 | 0 | 0 | 0 | 0 | 0 | 0 | 0 | 0 | 0 | 0 | 0 |
| 31 | F | 80 | 9 | 1.1 | 1.0 | 0 | 0 | 0 | 1 | 0 | 0 | 0 | 0 | 1 | 2 | 2 |
| 32 | F | 78 | 15 | 1.2 | 1.4 | 0 | 0 | 0 | 0 | 0 | 0 | 0 | 0 | 0 | 0 | 0 |
| 33 | F | 55 | 11 | 1.4 | 1.1 | 0 | 0 | 0 | 0 | 0 | 0 | 0 | 0 | 1 | 1 | 1 |
| 34 | M | 83 | 9 | 1.6 | 2.6 | 1 | 1 | 0 | 1 | 0 | 0 | 0 | 0 | 1 | 2 | 2 |
| 35 | F | 57 | 15 | 2.6 | 4.1 | 0 | 0 | 0 | 0 | 0 | 0 | 0 | 0 | 0 | 0 | 0 |
| 36 | F | 70 | 15 | 2.6 | 3.4 | 1 | 1 | 0 | 0 | 0 | 0 | 0 | 0 | 0 | 0 | 0 |
| 37 | M | 74 | 14 | 2.8 | 3.2 | 0 | 0 | 0 | 0 | 0 | 0 | 0 | 0 | 0 | 0 | 0 |
| 38 | F | 56 | 14 | 2.9 | 3.0 | 0 | 0 | 0 | 0 | 0 | 0 | 0 | 0 | 0 | 0 | 0 |
| 39 | M | 60 | 14 | 2.9 | 3.1 | 0 | 0 | 0 | 0 | 0 | 0 | 0 | 0 | 0 | 0 | 0 |
| 40 | F | 39 | 14 | 3.0 | 3.1 | 0 | 0 | 0 | 0 | 0 | 0 | 0 | 0 | 0 | 0 | 0 |
| 41 | M | 69 | 13 | 3.3 | 3.5 | 0 | 0 | 0 | 0 | 0 | 0 | 0 | 1 | 0 | 1 | 1 |
| 42 | M | 54 | 12 | 3.8 | 3.9 | 0 | 0 | 0 | 0 | 0 | 0 | 0 | 0 | 1 | 1 | 1 |
| 43 | M | 45 | 13 | 4.0 | 4.6 | 0 | 0 | 0 | 0 | 0 | 0 | 0 | 0 | 0 | 0 | 0 |
| 44 | M | 55 | 12 | 4.0 | 4.9 | 0 | 0 | 0 | 0 | 0 | 0 | 0 | 0 | 1 | 1 | 1 |
| 45 | M | 81 | 15 | 4.1 | 4.3 | 0 | 0 | 0 | 1 | 0 | 0 | 0 | 0 | 0 | 1 | 1 |
| 46 | M | 58 | 14 | 4.3 | 4.1 | 0 | 0 | 0 | 0 | 0 | 0 | 0 | 0 | 0 | 0 | 0 |
| 47 | M | 54 | 13 | 4.3 | 5.3 | 1 | 1 | 0 | 0 | 0 | 0 | 0 | 0 | 0 | 0 | 0 |
| 48 | F | 70 | 13 | 4.7 | 5.6 | 0 | 0 | 0 | 0 | 0 | 0 | 0 | 0 | 0 | 0 | 0 |
| 49 | M | 52 | 15 | 5.1 | 5.9 | 0 | 0 | 0 | 0 | 0 | 0 | 0 | 1 | 0 | 1 | 1 |
| 50 | M | 96 | 13 | 5.2 | 5.6 | 0 | 0 | 0 | 1 | 0 | 0 | 0 | 0 | 0 | 1 | 1 |
| 51 | M | 75 | 14 | 5.3 | 6.6 | 0 | 0 | 0 | 0 | 0 | 0 | 0 | 0 | 0 | 0 | 0 |
| 52 | M | 69 | 12 | 6.7 | 7.2 | 0 | 0 | 0 | 0 | 0 | 0 | 0 | 1 | 1 | 2 | 2 |
| 53 | M | 79 | 15 | 6.8 | 7.0 | 0 | 0 | 0 | 0 | 0 | 0 | 0 | 1 | 0 | 1 | 1 |
| 54 | F | 75 | 15 | 8.7 | 12.3 | 1 | 0 | 0 | 0 | 0 | 0 | 0 | 0 | 0 | 0 | 0 |
| 55 | M | 71 | 14 | 8.7 | 12.0 | 0 | 0 | 0 | 0 | 0 | 0 | 0 | 0 | 0 | 0 | 0 |
| 56 | M | 55 | 15 | 9.0 | 9.1 | 0 | 0 | 0 | 0 | 0 | 0 | 0 | 0 | 0 | 0 | 0 |
| 57 | F | 88 | 14 | 9.0 | 10.0 | 1 | 1 | 0 | 1 | 0 | 0 | 0 | 1 | 0 | 2 | 2 |
| 58 | M | 53 | 15 | 9.0 | 9.3 | 0 | 0 | 0 | 0 | 0 | 0 | 0 | 0 | 0 | 0 | 0 |
| 59 | M | 49 | 15 | 9.5 | 8.7 | 1 | 1 | 0 | 0 | 0 | 0 | 0 | 0 | 0 | 0 | 0 |
| 60 | M | 50 | 14 | 10.1 | 12.1 | 0 | 0 | 0 | 0 | 0 | 0 | 0 | 0 | 0 | 0 | 0 |
| 61 | F | 74 | 7 | 10.2 | 13.7 | 0 | 0 | 0 | 0 | 0 | 0 | 0 | 1 | 1 | 2 | 2 |
| 62 | M | 39 | 15 | 12.1 | 13.6 | 0 | 0 | 0 | 0 | 0 | 0 | 0 | 1 | 0 | 1 | 1 |
| 63 | M | 80 | 9 | 12.1 | 13.4 | 1 | 0 | 0 | 1 | 0 | 0 | 0 | 1 | 1 | 3 | 3 |
| 64 | F | 52 | 13 | 13.2 | 16.6 | 0 | 0 | 0 | 0 | 0 | 0 | 0 | 0 | 0 | 0 | 0 |
| 65 | M | 82 | 13 | 13.3 | 15.7 | 0 | 0 | 0 | 1 | 0 | 0 | 0 | 0 | 0 | 1 | 1 |
| 66 | M | 53 | 6 | 15.7 | 16.4 | 1 | 0 | 0 | 0 | 0 | 0 | 0 | 1 | 2 | 3 | 3 |
| 67 | M | 84 | 11 | 16.1 | 17.5 | 1 | 1 | 0 | 1 | 0 | 0 | 0 | 0 | 1 | 2 | 2 |
| 68 | M | 36 | 15 | 17.7 | 21.7 | 0 | 0 | 0 | 0 | 0 | 0 | 0 | 0 | 0 | 0 | 0 |
| 69 | M | 31 | 14 | 20.5 | 27.5 | 0 | 0 | 0 | 0 | 0 | 0 | 0 | 0 | 0 | 0 | 0 |
| 70 | M | 47 | 6 | 20.8 | 26.2 | 1 | 1 | 0 | 0 | 0 | 0 | 0 | 0 | 1 | 1 | 1 |
| 71 | F | 70 | 7 | 23.1 | 39.2 | 0 | 0 | 0 | 0 | 0 | 0 | 1 | 1 | 1 | 2 | 3 |
| 72 | M | 78 | 14 | 24.7 | 30.5 | 1 | 1 | 0 | 0 | 0 | 0 | 1 | 0 | 0 | 0 | 1 |
| 73 | F | 67 | 13 | 29.1 | 29.6 | 0 | 0 | 0 | 0 | 0 | 0 | 0 | 0 | 0 | 0 | 0 |
| 74 | M | 57 | 11 | 31.8 | 34.0 | 0 | 0 | 0 | 0 | 0 | 1 | 1 | 0 | 1 | 2 | 2 |
| 75 | M | 48 | 14 | 31.9 | 36.4 | 0 | 0 | 0 | 0 | 0 | 1 | 1 | 0 | 0 | 1 | 1 |
| 76 | M | 46 | 12 | 4.2 | 6.7 | 1 | 1 | 0 | 0 | 0 | 0 | 0 | 1 | 1 | 2 | 2 |
| 77 | F | 48 | 9 | 5.2 | 4.3 | 1 | 1 | 0 | 0 | 0 | 0 | 0 | 0 | 1 | 1 | 1 |
| 78 | M | 74 | 12 | 6.0 | 6.4 | 1 | 1 | 0 | 0 | 0 | 0 | 0 | 1 | 1 | 2 | 2 |
| 79 | M | 75 | 11 | 10.4 | 14.8 | 1 | 1 | 0 | 0 | 0 | 0 | 0 | 1 | 1 | 2 | 2 |
| 80 | M | 79 | 15 | 10.5 | 13.6 | 1 | 1 | 0 | 0 | 0 | 0 | 0 | 1 | 0 | 1 | 1 |
| 81 | M | 78 | 15 | 16.4 | 25.4 | 1 | 1 | 0 | 0 | 0 | 0 | 0 | 1 | 0 | 1 | 1 |
| 82 | F | 79 | 7 | 17.0 | 19.0 | 1 | 1 | 0 | 0 | 0 | 0 | 0 | 1 | 1 | 2 | 2 |
| 83 | F | 47 | 8 | 19.2 | 22.6 | 1 | 1 | 0 | 0 | 0 | 0 | 0 | 0 | 1 | 1 | 1 |
| 84 | M | 47 | 9 | 19.2 | 28.1 | 0 | 1 | 0 | 0 | 0 | 0 | 0 | 0 | 1 | 1 | 1 |
| 85 | M | 53 | 9 | 20.8 | 24.8 | 0 | 1 | 0 | 0 | 0 | 0 | 0 | 1 | 1 | 2 | 2 |
| 86 | F | 37 | 5 | 21.6 | 28.1 | 1 | 1 | 0 | 0 | 0 | 0 | 0 | 1 | 1 | 2 | 2 |
| 87 | M | 32 | 15 | 24.5 | 42.2 | 0 | 1 | 0 | 0 | 0 | 0 | 1 | 0 | 0 | 0 | 1 |
| 88 | F | 81 | 12 | 25.5 | 89.1 | 1 | 1 | 0 | 1 | 0 | 0 | 1 | 0 | 1 | 2 | 3 |
| 89 | F | 65 | 7 | 26.9 | 46.1 | 1 | 1 | 0 | 0 | 0 | 0 | 1 | 1 | 1 | 2 | 3 |
| 90 | M | 68 | 6 | 47.2 | 69.4 | 0 | 0 | 0 | 0 | 0 | 1 | 1 | 1 | 1 | 3 | 3 |
| 91 | F | 62 | 7 | 48.2 | 68.0 | 1 | 1 | 0 | 0 | 0 | 1 | 1 | 1 | 1 | 3 | 3 |
| 92 | M | 56 | 13 | 51.3 | 66.7 | 1 | 1 | 0 | 0 | 0 | 1 | 1 | 1 | 0 | 2 | 2 |
| 93 | M | 51 | 5 | 52.7 | 66.8 | 1 | 1 | 0 | 0 | 0 | 1 | 1 | 1 | 1 | 3 | 3 |
| 94 | F | 52 | 6 | 53.1 | 82.3 | 1 | 1 | 0 | 0 | 0 | 1 | 1 | 0 | 1 | 2 | 2 |
| 95 | M | 60 | 6 | 54.3 | 66.2 | 0 | 1 | 0 | 0 | 0 | 1 | 1 | 0 | 1 | 2 | 2 |
| 96 | F | 84 | 15 | 55.8 | 99.2 | 1 | 1 | 0 | 1 | 0 | 1 | 1 | 0 | 0 | 2 | 2 |
| 97 | F | 88 | 8 | 56.4 | 85.1 | 1 | 1 | 0 | 1 | 0 | 1 | 1 | 0 | 1 | 3 | 3 |
| 98 | M | 55 | 5 | 60.2 | 80.8 | 1 | 1 | 0 | 0 | 0 | 1 | 1 | 1 | 1 | 3 | 3 |
| 99 | M | 38 | 7 | 73.8 | 106.5 | 0 | 1 | 0 | 0 | 0 | 1 |  | 1 | 1 | 3 | 2 |
| 100 | M | 55 | 6 | 76.7 | 90.9 | 1 | 1 | 0 | 0 | 0 | 1 | 1 | 0 | 1 | 2 | 2 |
| 101 | M | 61 | 6 | 81.5 | 104.5 | 1 | 1 | 0 | 0 | 0 | 1 | 1 | 1 | 1 | 3 | 3 |
| 102 | M | 51 | 4 | 82.6 | 108.9 | 0 | 1 | 0 | 0 | 0 | 1 | 1 | 1 | 2 | 4 | 4 |
| 103 | M | 55 | 7 | 93.3 | 134.8 | 1 | 1 | 0 | 0 | 0 | 1 | 1 | 1 | 1 | 3 | 3 |
| 104 | M | 82 | 6 | 96.8 | 142.2 | 1 | 1 | 0 | 1 | 0 | 1 | 1 | 1 | 1 | 4 | 4 |
| 105 | M | 76 | 4 | 98.7 | 149.4 | 1 | 1 | 0 | 0 | 0 | 1 | 1 | 0 | 2 | 3 | 3 |
| 106 | F | 81 | 7 | 109.6 | 141.6 | 1 | 1 | 0 | 1 | 0 | 1 | 1 | 1 | 1 | 4 | 4 |

Note: Sex (M = male; F = female); age (year); volume (mL); Shape (1 = irregular; 0 = regular); expired (1 = expired; 0 = alive)
